# Effects of sublethal exposure to metofluthrin on the fitness of *Aedes aegypti* in a domestic setting in Cairns, Queensland

**DOI:** 10.1186/s13071-017-2220-7

**Published:** 2017-05-31

**Authors:** Tamara S. Buhagiar, Gregor J. Devine, Scott A. Ritchie

**Affiliations:** 1 College of Public Health, Medical and Veterinary Sciences, James Cook University, PO Box 6811, QLD, Cairns, 4870 Australia; 2 Mosquito Control Laboratory, QIMR-Berghofer Institute of Medical Research, QLD, Brisbane, 4006 Australia

**Keywords:** Metofluthrin, Spatial repellents, Pyrethroid-resistance, *Aedes aegypti*, Dengue

## Abstract

**Background:**

Metofluthrin is highly effective at reducing biting activity in *Aedes aegypti*. Its efficacy lies in the rapid onset of confusion, knockdown, and subsequent kill of a mosquito. In the field, there are a variety of scenarios that might result in sublethal exposure to metofluthrin, including mosquitoes that are active at the margins of the chemical’s lethal range, brief exposure as mosquitoes fly in and out of treated spaces or decreasing efficacy of the emanators with time. Sublethal effects are key elements of insecticide exposure and selection.

**Methods:**

The metofluthrin dose for each treatment group of male and female *Ae. aegypti* was controlled using *exposure time* intervals to a 10% active ingredient (AI) metofluthrin emanator. Room size and distance from the emanator for all groups was maintained at 3 m. In bioassay cages, male *Ae. aegypti* were exposed at 0, 5, 10, 20, 30 and 40-min intervals. Females were exposed in bioassay cages at 0, 10, 20, 30, 40 and 60-min intervals. Mortality rates and fecundity were observed between the *exposure time* groups for both sexes.

**Results:**

Female *Ae. aegypti* exposed for 60 min had a significantly higher mortality rate (50%), after a 24-h recovery period, than other exposure times, 10, 20, 30 and 40 min (*P* < 0.001). An overall difference in fecundity was not observed in females between treatments. A significant effect on male mortality was only observed at 40 min exposure times, three meters from the 10% AI emanator $\left(\overset{-}{X}=98\%,\kern0.5em  P<0.001\right)$. Males that survived metofluthrin exposure were as likely to produce viable eggs with an unexposed female as males that had not been exposed (*P* > 0.05).

**Conclusion:**

Regardless of sex, if a mosquito survived exposure, it would be as biologically successful as its unexposed counterpart. Portability of the metofluthrin emanator and delayed knockdown effects create opportunities for sublethal exposure and potential pyrethroid resistance development in *Ae. aegypti*, and should be taken into consideration in recommendations for field application of this product, including minimum exposure periods and a prescribed number of emanators per room based on volume.

## Background

The primary vector of dengue in north Queensland, and in most parts of the tropical regions of the world, is the yellow fever mosquito, *Aedes aegypti* (L.) [1, 2]. This day-biting mosquito has a close association with the domestic environment and is often described as an “urban mosquito” [3–5].

Mosquito coils and vaporizer mats are well-documented spatial repellents that have been used in repelling mosquitoes from human biting [6, 7]. Unfortunately, both of these products are limited by their requirement for a heat source to vaporize their active ingredient, efficacy [7, 8], and their limited use indoors [9]. Studies have shown varying susceptibility to pyrethroid-based mosquito coils between different mosquito species, and within a species, specifically *Ae. aegypti* [7, 8]. Increased efficacy of pyrethroid-based mosquito coils against *Ae. aegypti* is observed with the addition of a synergist [8]. Use of synergists, such as Octachlorodipropyl ether (S-2) are commonly used in Asia, but are illegal in the USA [9], as it exposes humans to some levels of bio-chloromethyl ether (BCME), an extremely potent lung carcinogen. A study by Liu et al., [10] found that burning one mosquito coil produced the same amount of fine and ultrafine particulate matter mass as burning 75–137 cigarettes. They also found a large suite of volatile organic compounds, including carcinogens and suspected carcinogens in the coil smoke [10].

Investigations into the insecticidal activity of norchrysanthemic acid esters with high vapor activity at ambient temperature [11] have resulted in the identification of an effective synthetic pyrethroid commonly known as metofluthrin. This compound does not require a heat source or burning of carcinogenic material, making it a suitable candidate for use indoors.

Metofluthrin is a volatile pyrethroid and has been shown to be extremely effective at reducing biting activity in *Aedes* species*,* including *Ae. aegypti*, as well as *Culex quinquefasciatus* in the field [12–14]. Metofluthrin has been shown not to have any repellent or expellant effects with the 5–10% active ingredient (AI) passive formulations used by Rapley et al. [6], and  Ritchie & Devine [15]. Biting reduction with metofluthrin is primarily achieved through the compound’s ability to confuse, knockdown, and kill *Ae. aegypti* [6, 15]. Due to its unique and effective mechanism for reducing biting, it could serve as an effective tool to disrupt biting by *Ae. aegypti* and potentially reduce dengue transmission [15].

Metofluthrin is a volatile pyrethroid that has insecticidal properties and well-documented behavioral effects on resting and biting [6, 15]. However, due to its insecticidal properties, portability, limits in spatial range, and delayed onset of knockdown and lethal effects, an opportunity is created for sublethal exposure of mosquitoes. In this study, we look at the impacts of sublethal exposure in semi-field conditions, controlled through *exposure time,* on adult *Ae. aegypti* fitness. To date, the physiological effects of sublethal exposure on *Ae. aegypti*, if any, have not yet been described. A study involving the exposure of *Ae. aegypti* to sublethal doses of deltamethrin, an insecticidal pyrethroid, found that unmated females were more likely to escape and less likely to blood feed than mated females in the presence of a host [16]. Cohnstaedt & Allan [17] found a significant reduction in the response of *Ae. aegypti*, *Culex quinquefasciatus*, and *Anopheles albimanus* to host attractants when exposed to a sublethal dose (LD_25_) of permethrin and deltamethrin for a 24 h period. Exposure of *Aedes vigilax* larvae to sublethal doses (LD_50_) of methoprene significantly reduced survival in adult females and males, and reduced blood-feeding success [18]. In a study by Wagman et al. [19], prolonged sublethal exposure of wild F_0_ *Ae. aegypti* that were not responsive to the repellent effects of transfluthrin demonstrated decreased susceptibility to transfluthrin toxicity by F_4_.

Methoprene’s insecticidal mechanism is inherently different from transfluthrin, permethrin, and deltamethrin, however, the principal remains the same. There are a variety of scenarios that might result in sublethal exposure to metofluthrin, including mosquitoes that are active at the margins of the chemical’s lethal range, periodic exposure as mosquitoes fly in and out of treated spaces or decreasing efficacy of the emanators with time. Sublethal effects are key elements of insecticide exposure and selection. In this study, we evaluate the impact of sublethal exposure to metofluthrin on the blood-feeding, mating success, and egg quality of adult *Ae. aegypti*. Determining the impacts of sublethal exposure on overall mosquito fitness and fecundity will highlight the potential for heritable insensitivity, as described by Wagman et al. [19] and pyrethroid resistance in wild, pyrethroid susceptible *Ae. aegypti*.

## Methods

**Fig. 1:**
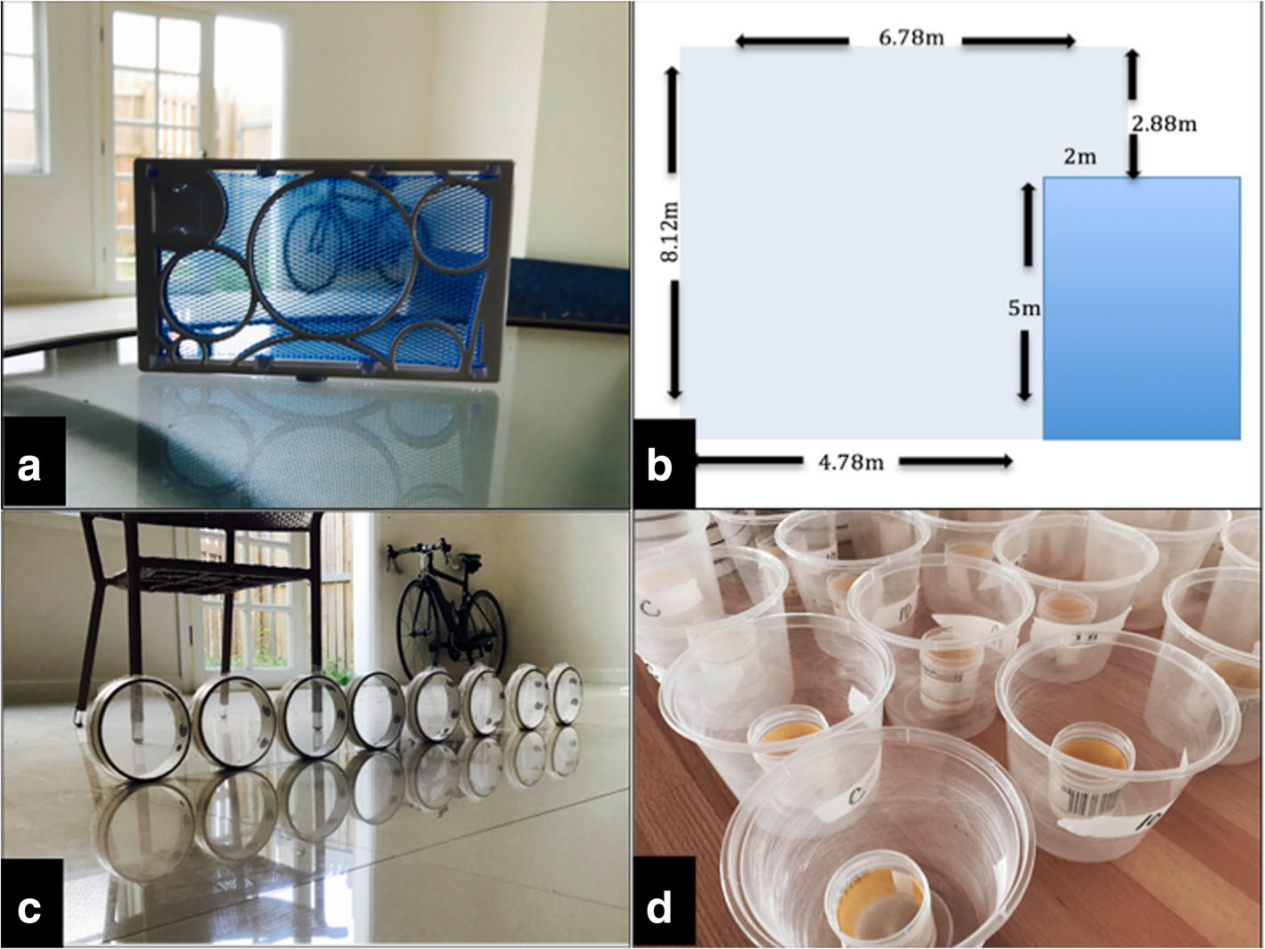
**a** 10% AI metofluthrin emanator (Sumitomo). **b** Floor plan of the experimental room*.* **c** Female mosquitoes in tambourine cages. **d** Containers where female cohorts were placed post-exposure

The metofluthrin product used in this study was a small (9.5 × 15 × 1 cm) plastic frame containing a polyethylene mesh in which the 10% AI formulation is incorporated into (Sumitomo Chemical Australia Pty Ltd., Sydney, Australia) [15] (Fig. 1a). In previous longevity studies of this emanator, its efficacy was sustained up to 20 days [15].

### Rearing, sexing, caging, and blood-feeding mosquitoes

A Cairns colony (F1) of *Ae. aegypti* infected with *w*Mel *Wolbachia,* derived from field populations, was used throughout the experiments. The colony was reared in a controlled temperature room at 25 °C and maintained at 70% relative humidity. *Wolbachia*-infected mosquitoes were used to avoid accidental introduction of uninfected mosquitoes in an area where the Eliminate Dengue program had established *w*Mel in *Ae. aegypti.* In the experiments requiring virgin females, mosquitoes were separated as pupae by size and placed in cohorts of ten. Once they had emerged, and it was certain that the females were not exposed to a male, they were then placed into a BugDorm (30 × 30 × 30 cm) with other virgin females, and provided with a 10% honey solution. When mosquitoes were required to take a blood meal, honey pads were removed 24 h prior. When blood meals were required, mosquitoes were offered a blood meal by resting the back of a human leg on top of the cage or container (Human Ethics Approval from James Cook University, H6286).

### Experimental setting

All experiments took place within a large living room (111 m^3^) within a Queenslander-style house in Cairns, Queensland, Australia (Fig. 1b). The 10% AI metofluthrin emanator was placed centrally within the room and allowed to volatilize for 30 min prior to the commencement of each experimental repetition.

### Effects on fitness and fecundity: females

Cohorts of approximately 10 mated female *Ae. aegypti* were placed into fine mesh tambourine cages (Fig. 1c) and exposed in the metofluthrin-treated room at 3 m from the emanator for 10, 20, 30, 40 and 60 min. The control group, 0 min, was kept in an unexposed room within the same dwelling. Once exposed, the knockdown rate was recorded for each exposure time. Each female cohort was then placed into 500 ml plastic container. The containers were roughened on the inside to ensure the mosquito could easily rest on the walls. Inside the containers, was a smaller 100 ml cup with a sand paper egg-laying strip, half-submerged in water. The containers were covered with a muslin cloth. The containers were transparent, and mosquitoes could be easily observed without being removed (Fig. 1d).

Mortality was observed after 3 and 24 h post-exposure. A blood meal was offered to each cohort at 24 h for 5 min, and the proportion of blood-fed mosquitoes was recorded. The egg strips were removed 7 days after blood meal, and stored for 72 h to embryonate. The eggs were then immersed in water with yeast to induce hatching. After 48 h, the egg strips were examined under a microscope to determine the displacement of the operculum from the egg, an indicator that the egg was viable and had hatched. The mean number of eggs laid per blood-fed female for each treatment and the mean hatch rate was recorded. A total of 6 replicates were completed.

### Effects on fitness and mating success: males

Cohorts of 5 virgin male *Ae. aegypti* were placed into tambourine cages and exposed to metofluthrin for 5, 10, 20, 30 and 40 min at 3 m from the emanator. The control group, 0 min, was kept in an unexposed room within the same dwelling. The knockdown rate was recorded for each treatment. Mortality was observed after 6 h. Surviving individual males were placed individually into a single plastic 250 ml cup containing one virgin female. Each female had previously been blood-fed 3 days prior. Inside each cup was a small 100 ml container with a sand paper egg-laying strip, half-submerged in water. The 250 ml cups were then covered with a muslin cloth, and mosquito pairs were given 7 days to mate and produce eggs on the oviposition strip. Eggs laid onto the strip were removed from the water and stored for 72 h to embryonate, then immersed in water with yeast to induce hatching. After 48 h, egg strips were examined under a microscope to determine the displacement of the operculum from the egg, an indicator that the egg was viable and had hatched. The number of eggs laid and the proportion of eggs hatched were recorded for each male/female pair.

### Data analyses

#### Effects on fitness and fecundity: females

Knockdown and mortality data were arcsine square root transformed and analyzed using a 2-Way ANOVA followed by a Tukey’s multiple comparisons test. Blood-feeding and fecundity data were arcsine square root transformed and analyzed using a One-Way ANOVA followed by a Dunnett’s multiple comparisons test.

#### Effects on fitness and mating success: males

Mortality and mating success data were arcsine square root transformed and analyzed using a One-Way ANOVA. A Holm-Sidak’s multiple comparisons test for the mortality data was performed. Hatch rate data were analyzed using a One-Way ANOVA, followed by a Dunnett’s multiple comparisons test.

All data were analyzed in Prism 6 for Mac OSX (v. 6.0 h, GraphPad Software Inc.).

## Results

### Females

#### Knockdown and mortality

**Fig. 2:**
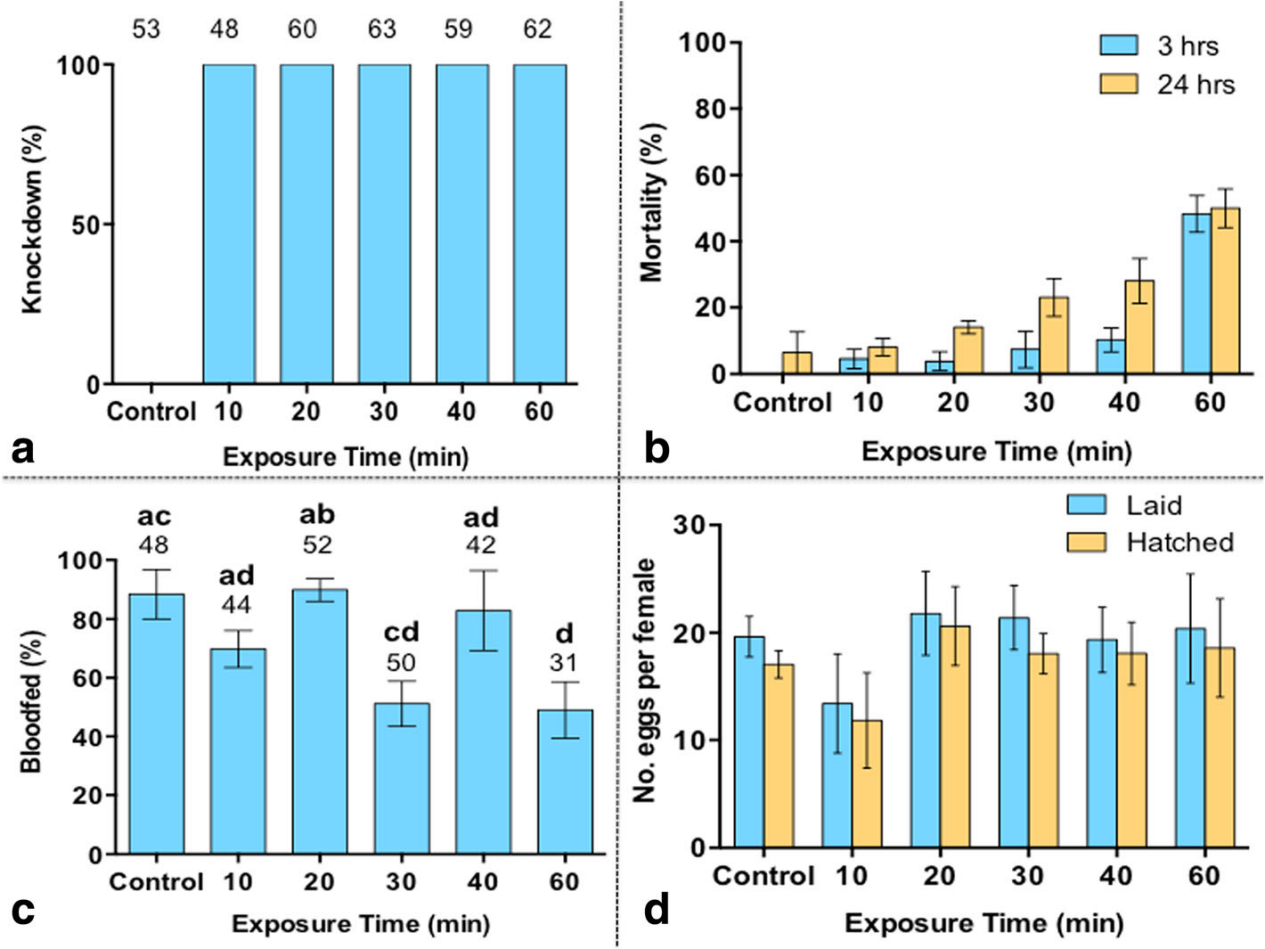
**a** Mean percent ± SE knockdown of female *Ae. aegypti* exposed to metofluthrin (10% AI). The number above each column represents the total number of females exposed in each treatment group (*n* = 6). **b** Mean percent ± SE mortality of females 3 and 24 h post-exposure to metofluthrin. **c** The mean percent ± SE of blood-fed mosquitoes at 24 h. **d** Mean percent ± SE of eggs laid and hatched per treatment. There was no significant effect of treatment on the number of eggs laid per female or the hatch rate of those eggs

One hundred percent of all female *Ae. aegypti* exposed to metofluthrin (10%) for 10 min, or more at 3 m from the emanator were knocked down (Fig. 2a). Forty-eight per cent of females exposed for 60 min were dead at 24 h post-exposure. At 3 and 24 h post-treatment, a 2-Way ANOVA found a significant effect of exposure time on mortality, and a significant effect of the amount of time post-exposure on mosquito mortality (*F*_(5,30)_ = 35.67, *P* < 0.0001 and *F*_(1,30)_ = 15.13, *P* < 0.001, respectively) (Fig. 2b). A Tukey’s *post-hoc* analysis found that at 3 h post-treatment, a significant difference in the effect of metofluthrin on mortality was observed between the 60-min exposure treatment and all other treatment groups (*P* < 0.0001). At 24 h post-exposure, a significant difference in mortality was observed between the control and mosquitoes exposed for 30 min or more (*P* < 0.001). After 24 h post-exposure, 50% of female *Ae. aegypti* survived exposure and received a sublethal dose of metofluthrin after 60 min.

#### Blood-feeding and fecundity

Female *Ae. aegypti* that survived metofluthrin exposure between 10 and 60 min were considered *sublethally exposed* to metofluthrin (10% AI) after a 24 h recovery period. A One-Way ANOVA of transformed blood-feeding data from sublethally exposed females found an overall significant effect of treatment (duration of exposure to metofluthrin) on blood-feeding success (*F*_(5,30)_ = 4.977, *P* = 0.002). A Dunnett’s *post-hoc* analysis found a significant difference between the control group and mosquitoes sublethally exposed for 30 and 60 min (Fig. 2c). A One-Way ANOVA of transformed egg hatching data, found no significant effect of treatment on the fecundity of sublethally exposed females (*F*_(5,28)_ = 1.33, *P* = 0.28). Female *Ae. aegypti* that survived metofluthrin exposure were as likely to lay viable eggs as each other and as the control (Fig. 2d).

### Males

#### Knockdown and mortality

**Fig. 3:**
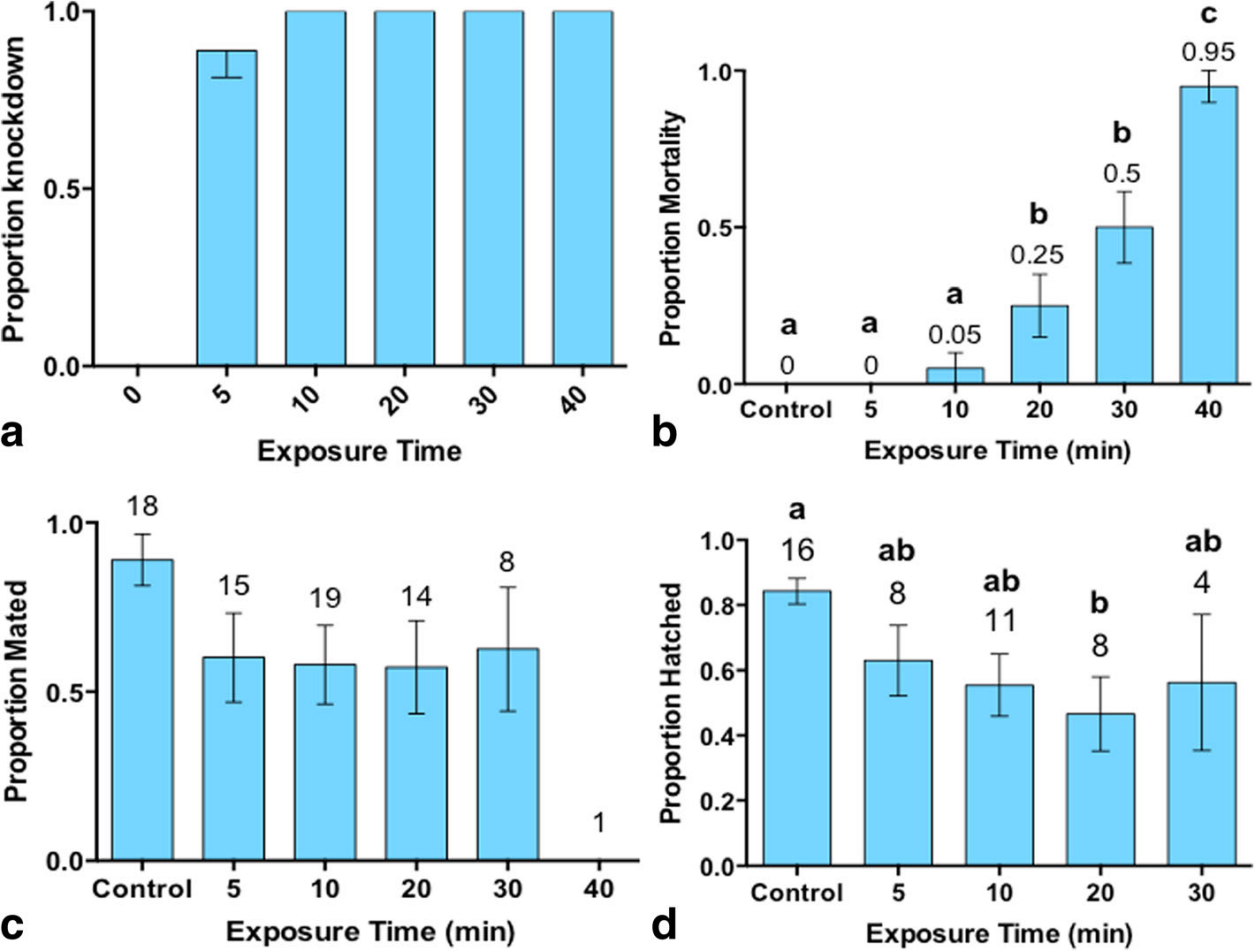
**a** Mean ± SE knockdown rates of male *Ae. aegypti* exposed to metofluthrin. **b** Mean ± SE mortality of male *Ae. aegypti* exposed to metofluthrin (10% AI) for 0, 5, 10, 20, 30 and 40 min exposure periods. Survival significantly decreases after 20 min of exposure. **c** Mean ± SE proportion of males that survived metofluthrin exposure and mated successfully. Mating success was confirmed by the production of eggs by the paired female. The number above each column is the *n*-value. No significant difference in mating success was observed between groups. **d** Mean proportion ± SE of eggs that hatched for each successfully mated male/female pair. The number above each column is the *n*-value

One hundred percent of all male *Ae. aegypti* exposed to metofluthrin (10%) for 10 min or more were knocked down (Fig. 3a). A One-way ANOVA of transformed data found an overall significant effect of treatment on mortality rates amongst treatment groups (*F*_(5,18)_ = 5.415, *P* = 0.0033) (Fig. 3b). A Holm-Sidak’s multiple comparisons test found no significant difference in mortality between the control ($\overset{-}{X}=0\%\Big)$ and males exposed up to 30 min (Fig. 3a). However, a significant effect of treatment on mortality was observed between the control ($\overset{-}{X}=0\%\Big)$ and males exposed for 40 min ($\overset{-}{X}=98\%\Big)$ (*P* < 0.001) (Fig. 3b).

#### Mating success

Males that survived metofluthrin exposure between 5 and 40 min were considered *sublethally exposed*. A One-Way ANOVA found no significant effect on mating success between treatment groups (*F*_(5,70)_ = 1.915, *P* = 0.103) (Fig. 3c), indicating that regardless of exposure time if a male *Ae. aegypti* survived metofluthrin exposure, he was as likely to successfully mate as an unexposed male when paired with an unexposed female. A One-Way ANOVA of transformed egg hatching data found an overall significant effect of treatment on egg viability, measured by the hatch rate of the eggs (*F*_(4,41)_ = 3.183, *P* = 0.0225). A Dunnett’s multiple comparisons analysis found a significant difference in egg viability, as measured by the proportion hatched, solely between the control group $\left(\overset{-}{X}=0.84\right)$ and the 20-min exposure treatment $\left(\overset{-}{X}=0.47\right)$ (Fig. 3d).

## Discussion

Metofluthrin causes a reduction in human biting through two main modes of pyrethroid action, firstly as a result of its knockdown effects and secondly due to the disruption in orientation towards the host [14]. Ritchie & Devine [15] found that exposure to 10 % AI emanators within small rooms rapidly affected all free-flying *Ae. aegypti*, reducing human landing counts to “negligible” after eight minutes, and after 20 min, 90% of mosquitoes were dead. Rapley et al. [6] found 98% mortality of *Ae. aegypti* in the presence of metofluthrin (4.1% AI) after 24 h. In this study, we did not observe the same impacts on mosquito survival at 20 min as described in Ritchie & Devine with the same emanator formulation [15]. We believe that this can be accounted for as a result of room size and the exposure of mosquitoes to metofluthrin from within a cage. In this study, the size of the room was 111 m^3^versus 22.3 and 24.3 m^3^ in those used by Ritchie & Devine [15]. In larger room studies using bioassay cages, effects of metofluthrin (10% AI) on mortality in female *Ae. aegypti* exposed within one meter of the emanator, were not observed until one hour of exposure in rooms that were 41.2 and 37.8 m^3^ [20]. The mosquitoes used in Ritchie & Devine [15] were also free flying compared to caged mosquitoes used in Darbro et al. [20] and in the set of experiments undertaken here. The fine mesh of the cages may have provided some protection by restricting airflow into the cage [15, 20]. Bibbs & Xue [21] also found limited effective range in a 31.2% concentration OFF! Clip-on repellent device used outdoors, whereby significant knockdown and mortality effects were not sustained beyond 0.3 m in caged *Ae. aegypti*. Although “kill” is a desirable outcome as a result of metofluthrin exposure, it is its immediate impacts on host-seeking activity that is of most importance to the host.

In terms of insecticide resistance, the insecticidal properties of metofluthrin become very important. Evaluating the potential for sublethal exposure of mosquitoes to occur when metofluthrin is used in practice, combined with determining any measurable impacts or lack thereof on *Ae. aegypti* fitness is essential when determining “best practice” for field application of this product. Insecticides in low or insufficient doses have been shown to have sublethal effects on mosquito fitness, and behavior [16, 18], as well as have significant impacts on sensitivity and toxicological vulnerability of the mosquito in subsequent generations [19]. Sublethal conditions were created in these experiments by controlling the exposure time of the mosquitoes and maintaining their distance from the emanator. A mosquito that is not near enough to the emanator or not exposed for a long enough period to be killed can be defined as “sublethally exposed.” Excluding sublethal studies from the evaluation of an insecticide could mean that the overall efficacy of a product, when applied, could be greatly underestimated.

Male *Ae. aegypti*, overall, are more vulnerable than females to metofluthrin exposure, as reflected in their mortality rates, however unlike females, they only need to survive a relatively short period of time, enough to find a female and mate, completing the single act required of that individual to pass its genetic material on to the next generation. *Aedes aegypti* females, when given the opportunity to recover from knockdown, will, once out of the effective range of an emanator, resume blood-feeding, mating, and ovipositioning with the same success as an unexposed individual. This opportunity to recover could result if, for example, the metofluthrin emanator is removed from the room or a draft blows the knocked down mosquito out of the effective range. Regardless of sex, if a mosquito survived exposure, it was as biologically successful as its unexposed counterpart. Sublethal exposure, as observed in this suite of experiments, appears to have no obvious effect on the fitness of *Ae. aegypti,* but should be considered in its recommendations for use and deployment*.*

Metofluthrin-treated devices deliver protected zones. In this experiment specifically, we placed the cages at three meters distance from the emanator, and observed 100% knockdown within ten minutes of both males and females, rendering them incapable of flying or biting. Its at the margins of the defined effective zones of the metofluthrin emanator, where mosquitoes will be less affected and may fully recover if they are able to orientate away from the chemical source or if the treatment is removed. Since metofluthrin is a confusant, rather than a repellant, this may be a minor issue as it does not expel the mosquito from the treated area, as observed in Ritchie & Devine [15]. As demonstrated in previous experiments using metofluthrin emanators, mosquitoes entering the effective zone of metofluthrin, are likely to experience a rapid onset of negative effects, significantly affecting their fitness, including flight, host seeking, and biting. These immediate effects on flight and host orientation, in combination with metofluthrin’s lack of repellant or expellant properties [15], may be conducive to the mosquito remaining in the effective range, eventually succumbing to its insecticidal effects, particularly indoors. Environments, where sublethal exposure is most likely to occur, are semi-enclosed areas, for example, a covered veranda or a laundry area attached to the home where mosquitoes are able to move out of the effective range more easily.

This study involved controlled exposure of *Ae. aegypti* to a series of metofluthrin *doses* determined by exposure time to the metofluthrin emanator, while distance of the mosquitoes from the emanator and the size of the room was maintained. Mortality rates for a series of *doses* provide a guideline for minimum exposure periods required for sufficient insecticidal impacts on *Ae. aegypti* in the field within an enclosed space*.* Future investigations involving metofluthrin should be directed toward determining metofluthrin’s ability to cause resistance in pyrethroid-susceptible populations *of Ae. aegypti,* defining its effective range, and development of usage and deployment protocols that maximize its insecticidal impacts, thus reducing opportunities for sublethal exposure.

## Conclusion

Overall, *Ae. aegypti* that survive exposure to metofluthrin (sublethal exposure) will be as biologically successful as their unexposed counterparts. Metofluthrin is an insecticide that volatilizes at room temperature, is portable, has a limited spatial range, and delayed knockdown and lethal effects. Portability of the product and delayed knockdown effects create opportunities for sublethal exposure and potential pyrethroid resistance development in *Ae. aegypti*. The results of our study combined with the described attributes of the metofluthrin emanator should be taken into consideration in recommendations for field application of this product, including minimum exposure periods and a prescribed number of emanators per room based on volume.

## Abbreviations

AI: Active ingredient
SE: Standard error
